# Does HbA1c Influence the Relationship between Stress and Cognition? Findings from the VA Normative Aging Study

**DOI:** 10.1093/geroni/igab046.2621

**Published:** 2021-12-17

**Authors:** Austin Brockmann, Carolyn Aldwin, Avron Spiro

**Affiliations:** 1 Oregon State University, Corvallis, Oregon, United States; 2 Boston University, Boston, Massachusetts, United States

## Abstract

Type 2 diabetes has increased in prevalence globally, with potential adverse effects on cognition. Both high levels of hemoglobin A1c (HbA1c) and stressful life events (SLEs) are associated with impaired cognitive performance, but few studies have examined their synergistic effects. The present study examined direct effects of stress and HbA1c on several cognitive outcomes, and whether HbA1c moderated the relationship between SLEs and cognition. Utilizing a sample of 527 older men from the VA Normative Aging Study (Mage = 74.3, SD = 6.5), stress was inversely related to MMSE, verbal fluency, and pattern recognition; HbA1c was only inversely associated with MMSE. The moderation model was supported only for pattern recognition (β = 1.64, p < .05), with stress having worse effects in those high in HbA1c. Stratifying analyses by age group (<75, 75+) showed that stress predicted cognition only in the young-old, while HbA1c was inversely related to cognition only in old-old participants. Further, these age-group analyses yielded different effects of demographics on cognition. In the young-old, age was consistently inversely related to all cognitive outcomes, but in the old-old only with MMSE and word list recall. Among the young-old, education was associated with only word list recall but improved performance for most scales among the old-old. Finally, HbA1c intensified the effect of stress moderation on verbal fluency only in old-old (β = 2.78, p < .05). In summary, stress was more important for cognition in the young-old, while education and health status were more important in the old-old.

